# Serum Mg, Zn and Fe levels among coronary artery disease patients in an urban south Indian region

**DOI:** 10.6026/973206300200070

**Published:** 2024-01-31

**Authors:** G Lakshmi Swetha, Reshma Devarajachar, Harish Rangareddy, Chethana Chethan, H Srinivas

**Affiliations:** 1 Department of Community Medicine, Sapthagiri Institution of Medical Sciences, Bangalore, India; 2 Department of Biochemistry, BGS Global Institute of Medical Sciences, Bangalore, India; 3 Department of Biochemistry, Haveri Institute of Medical Sciences, Haveri, India; 4 Department of Biochemistry, BGS Global Institute of Medical Sciences, Bangalore, India; 5 Department of Biochemistry, BGS Global Institute of Medical Sciences, Bangalore, India

**Keywords:** trace elements, oxidative stress, coronary artery disease

## Abstract

Nutrition plays a crucial role in CAD development, with trace elements like zinc, magnesium, copper, and iron impacting atherogenesis through their antioxidant or oxidant activity. This cross sectional study was conducted under the ICMR-STS program with IEC approval with the aim to estimate and correlate serum magnesium, zinc, and iron levels in CAD patients compared to healthy Individuals in the Urban South Indian population (50 cases, 50 controls, aged 40-70 years). Statistical analyses revealed a significant difference in serum iron levels between cases (95.10 ± 38.82 µg/dL) and controls (118.30 ± 50.54 µg/dL) with a p-value of 0.012. Serum magnesium levels showed a marginal difference between cases (1.97±0.11 mg/dL) and controls (1.92±0.15 mg/dL) with a p-value of 0.053. However, serum zinc levels did not exhibit a statistically significant difference between cases (123.47 ± 26.35 mg/dL) and controls (118.90 ± 32.77 mg/dL) with a p-value of 0.445. Thus, data shows the association between low serum iron levels and an increased risk of coronary artery disease.

## Background:

Coronary artery disease (CAD) remains the primary cause of global mortality and morbidity across genders [1]. The pathogenesis of cardiovascular disorders involves numerous risk factors, with micronutrients and trace elements playing a pivotal role. Despite its association with increased CAD risk, magnesium demonstrates beneficial effects on traditional CAD risk factors viz., hypertension, diabetes mellitus and dyslipidemia [2]. However, the direct relationship between serum/dietary magnesium and CAD risk remains inconclusive across different ethnic groups [3]. Magnesium deficiency has also been attributed to the causation of arrhythmias in acute myocardial infarction patients [4]. A decreased zinc level is associated with CAD in specific populations [5]. Reduced serum zinc levels have been reported in CAD patients, and zinc deficiency is recognized as a risk factor for ischemic heart disease [6,7]. Zinc deficiency is an indicator of poor prognosis in CAD [8]. Limited research on the relationship between zinc deficiency and CAD exists in the Indian population [9]. Iron, an essential trace element, is crucial for cellular processes but can be toxic in excess. Excessive iron may generate reactive oxygen species, contributing to oxidative stress implicated in various pathologies, including cardiovascular diseases [10]. Atherosclerosis, a multifactorial disease, involves factors such as endothelial dysfunction, vascular inflammation, and the accumulation of lipids within arterial walls. Traditional risk factors, including hypertension, diabetes, age, sex, obesity, family history, and smoking, contribute to atherosclerosis by increasing the production of free radicals [11]. Along with the theory of oxidative stress, atherosclerosis is the consequence of the oxidative modification of low density lipoproteins (LDL) in the arterial wall by reactive oxygen species (ROS) [12]. While strong evidence supports the involvement of oxidative free radicals in the development of degenerative diseases like CAD, conclusive proof remains elusive despite ongoing research into the roles of trace elements and their essential mechanisms in human life [13]. Trace elements zinc, magnesium, copper and iron are micronutrients with known antioxidants and/or oxidant activity it is pertinent to assess their role in CAD [14]. Therefore, it is of interest to document the serum magnesium, zinc, and iron levels in coronary artery disease patients in an urban south Indian population.

## Methodology:

The study design is a cross-sectional approach with purposive sampling, enrolling 50 individuals diagnosed with coronary heart disease (40-70 years) as cases, alongside 50 age and gender-matched healthy controls. Written informed consent was obtained from all participants prior to their inclusion. The sample size was determined using a formula for comparing means between two independent groups, considering a 5% type one error rate, 80% statistical power, and a standardized effect size (Δ = 0.8) for the main study outcomes, resulting in a total of 50 samples. In the biochemical analysis phase, 3mL of blood was drawn from each participant for serum separation, and the following estimation methods were employed: serum Magnesium by Xylidyl Blue method, serum Zinc by Nitro-PAPS method, and serum Iron by Ferrozine method.

## Statistical analysis:

The collected data were tabulated in Microsoft Excel, and subsequent statistical analyses were performed utilizing OpenEPI info software. The normal distribution of data was assessed through the Kolmogorov-Smirnov test, confirming a normal distribution. Subsequently, the Student's "t" test was employed to compare means, and Pearson's correlation analysis was conducted to elucidate the relationship between micronutrients in CAD. Data was represented as Mean ± SD.

## Results:

**Figure 1:**
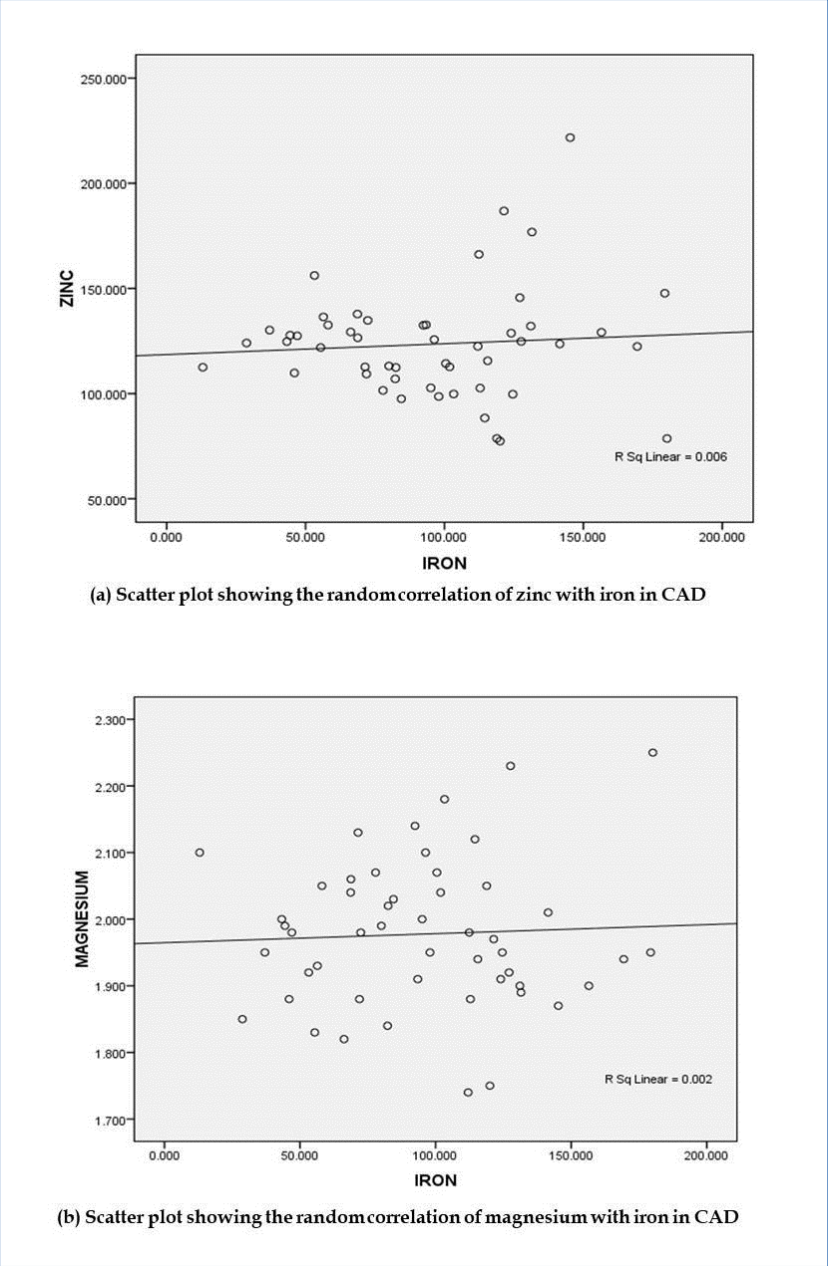
Pearson's correlation of serum zinc (a) and magnesium (b) with serum iron

The study comprised 100 participants, categorized into two groups: 50 individuals diagnosed with Coronary Artery Disease (CAD) from our hospital, and 50 healthy controls. The mean age for controls was 48.36±15.08 years, whereas the cases had a mean age of 55.86±9.91 years. A male preponderance of CAD was evident with a male-to-female ratio of 3.2:1 (male n=38, female n=12). A significant difference was observed in serum iron levels between cases (95.10 ± 38.82 µg/dL) and controls (118.30 ± 50.54 µg/dL) with a p-value <0.05 as shown in Table 1. Serum magnesium levels showed a marginal difference between cases (1.97±0.11 mg/dL) and controls (1.92±0.15 mg/dL) with a p-value of 0.053. However, serum zinc levels did not exhibit a statistically significant difference between cases (123.47 ± 26.35 mg/dL) and controls (118.90 ± 32.77 mg/dL) with a p-value of 0.445. Notably, a positive correlation was observed between iron and zinc, as well as iron and magnesium in CAD, as illustrated in Figure 1. However, the correlation was not statistically significance. These findings warrant further exploration, given the existing dearth of data regarding the elevation of these elements in the context of CAD.

## Discussion:

Nutrition plays a crucial role in cardiovascular disorders, with trace elements like zinc, magnesium, copper, and iron exhibiting known antioxidant or oxidant activity and influencing atherogenesis in coronary artery disease (CAD). In this study, we assessed the levels of iron, zinc, and magnesium in patients with CAD and apparently healthy subjects. Our findings revealed a higher level of serum zinc in CAD patients compared to healthy controls, consistent with literature suggesting that intracellular zinc release, triggered by events such as ischemia and infarction, can elevate serum zinc levels, as observed in our study [15]. However, conflicting reports indicate a decrease in serum zinc concentration after myocardial infarction [16]. Unlike studies reporting a decline in serum zinc within 24-48 hours post-event [17], our results did not show a significant fall, likely due to our sample collection occurring outside the acute phase of the critical event. Low serum iron is associated with cardiovascular disease [18]. Our study results identified significant changes in serum iron levels between CAD patients and controls, with CAD patients exhibiting low serum iron levels. This aligns with findings of increased iron stores in CAD by Pourmoghaddas *et al*. [19]. In contrast Bagheri *et al*. concluded that serum iron is elevated in atherosclerotic heart disease and correlates with its severity [20]. Human body has evolved a delicately balanced network to monitor iron entry, transport it to sites of need, and serve as a distinctive storage and recycling system, in the absence of an excretory system, to remove excess iron through intestinal absorption and shedding [21]. However studies have found that stored iron concentrations, as assessed by serum ferritin, is a strong and independent risk factor for premature CAD [22]. Excess iron, with its ability to generate reactive oxygen species, is implicated in oxidative stress and organic biomolecule oxidation [10]. High serum ferritin levels have been linked to an increased risk of atherosclerosis in the absence of other risk factors, catalyzing oxygen free radical production and lipid peroxidation, ultimately leading to oxidized LDL formation [23]. Magnesium is essential for ATP activation necessary for the sodium-potassium pump maintenance, and magnesium deficiency has been associated with arrhythmias in acute myocardial infarction patients [24]. Studies have shown reduced serum magnesium levels in patients with acute myocardial infarction and ischemic heart disease [24,25]. In our study, magnesium values were within the normal range, possibly attributed to the timing of sample collection in the second week post-event. These findings underscore the complex interplay of trace elements in cardiovascular health and warrant further investigation.

## Conclusion:

Serum zinc and iron levels may experience elevation in response to increased oxidative stress in coronary artery disease (CAD). Additionally, the observed elevation in serum magnesium levels in CAD could potentially be attributed to its role as a cofactor of creatinine phosphokinase, given the increased activity of this enzyme in CAD. Nevertheless, it is crucial to acknowledge that CAD has a multifactorial etiology, and even simple biochemical markers may prove valuable in predicting the risk of CAD and its associated complications. Further research is warranted to elucidate the intricate mechanisms underlying these biochemical changes and their implications in the context of CAD pathogenesis.

## Funding sources:

No funding was received for this research.

## Figures and Tables

**Table 1:** Comparison of serum iron, magnesium and zinc among study groups

**Parameter**	**Cases (n=50) Mean ± SD**	**Controls (n=50) Mean ± SD**	**t value**	**p value**
Serum Iron (µg/dL)	95.10 ± 38.82	118.30 ± 50.54	2.57	0.012*
Serum Magnesium (mg/dL)	1.97 ± 0.11	1.92 ± 0.15	-1.95	0.053
Serum Zinc (µg/dL)	123.47 ± 26.35	118.90 ± 32.77	-0.768	0.445
